# Early stage effect of ischemic preconditioning for patients undergoing on-pump coronary artery bypass grafts surgery: systematic review and meta-analysis

**DOI:** 10.12669/pjms.303.4292

**Published:** 2014

**Authors:** Qing Chai, Jin Liu

**Affiliations:** 1 Qing Chai, PhD, Department of Critical Medicine and Anesthesiology, West China Hospital, Sichuan University, Sichuan Province, China.; 2 Jin Liu, MD, Department of Critical Medicine and Anesthesiology, West China Hospital, Sichuan University, Sichuan Province, China.

**Keywords:** Ischemic heart disease, ischemia preconditioning, ischemia/reperfusion injury, Coronary artery bypass grafts, Myocardial protection

## Abstract

***Background:*** During the on-pump coronary artery bypass grafts surgery, ischemia/reperfusion injury would happen. Ischemia preconditioning could increase the tolerance against subsequent ischemia and reduce the ischemia/reperfusion injury. However the clinical outcomes of the available trials were different.

***Methods*** ***: ***We searched the Cochrane Central Register of Controlled Trials on The Cochrane Library (Issue 3, 2013), the Medline/PubMed and CNKI in March 2013. RevMan 5.1.6 and GRADEprofiler 3.6 were used for statistical analysis and evidence quality assessment. Heterogeneity was evaluated with significance set at P≤0.10.

***Results: ***Eighteen randomized controlled trials were included. There were no differences on in-hospital mortality, postoperative myocardial infarction morbidity between ischemia preconditioning and control groups. The heterogeneity of creatine kinase-MB level 24 hours after surgery was obvious. The differences of 72 hours area under the curve of cardiac troponin T (mean differences of -14.50, 95% confidence interval of -21.71 to -7.28) and troponin I (mean differences -181.79, 95% confidence interval of -270.07 to -93.52) after surgery were observed.

***Conclusion*** ***s*** ***:*** All the 18 trails, the positive and the negative results were equal. The meta-analysis results should be interpreted with caution due to limited effective data. Because of high cost-effectiveness, ischemia preconditioning could not be denied completely. Large-scale randomized studies are needed, with the operation procedures and included criteria being more specific.

## INTRODUCTION

Coronary heart disease (CHD) causes a severe health burden in the world. An estimated of 7.3 million people died from CHD in 2008. Over 80% of deaths take place in low- and middle-income countries and occur almost equally in men and women. On-pump coronary artery bypass grafts surgery (CAGB) is one of the main treatments for CHD.^1^ During the surgery process, ischemia/reperfusion injury (IRI) would happen and causes the injury of heart. In order to reduce the adverse effects of IRI, many regimens have been investigated.

The concept of ischemia preconditioning (IP) was first identified by Murry et al^2^ in 1986. It emerged that a brief episode of intermittent IRI could activate intrinsic protective mechanism which increased the tolerance against subsequent critical ischemia and reduced the IRI. Then the protective function against. IRI by brief periods of ischemia at a remote site beyond the target organ was firstly observed by Przyklenk et al^3^ in 1993. This was named as remote IP (RIPC). IP has an early effect (also known as first window effect) and a late effect (also known as second window effect). The early effect occurs within several minutes after the stimulus and lasts for about six hours, while the late effect occurs within 24 hours after the stimulus and lasts for about 96 hours. Over the past 26 years, scientific researchers have exploited much about the biology and underlying mechanism of IP, and it has been expanded to many organ systems in relevant scenarios.

Until now a large number of experiments have been performed to testify the validity of IP. Most of them were focus on heart protection. However, according to the available small trials, the clinical outcomes were different. The necessity of IP for clinical patients is still controversial. Therefore we pooled the clinical data and conducted a meta-analysis and systematic review to examine that whether patients with on-pump CABG surgery could get benefits from IP.

**Fig.1:**
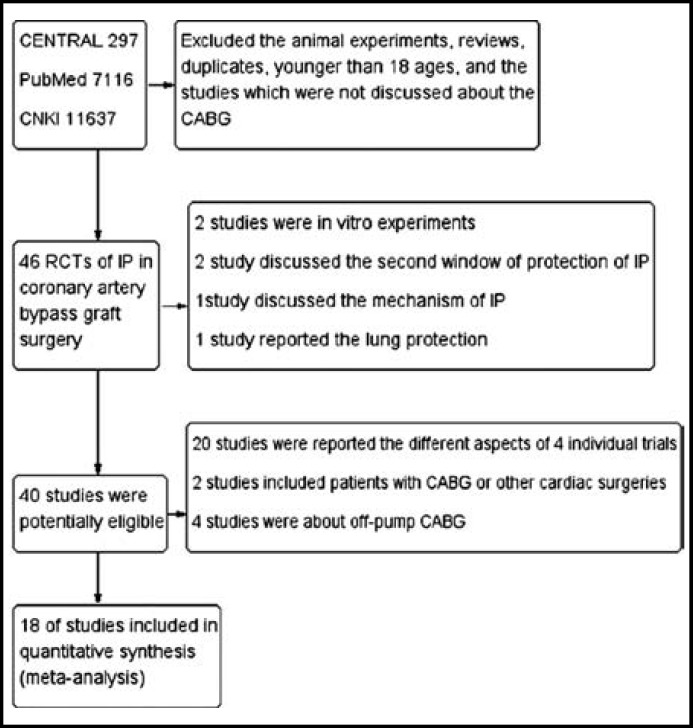
Flow diagram (CABG: coronary artery bypass graft surgery; RCT: randomised controlled trials; IP: ischemic preconditioning).

**Fig.2:**
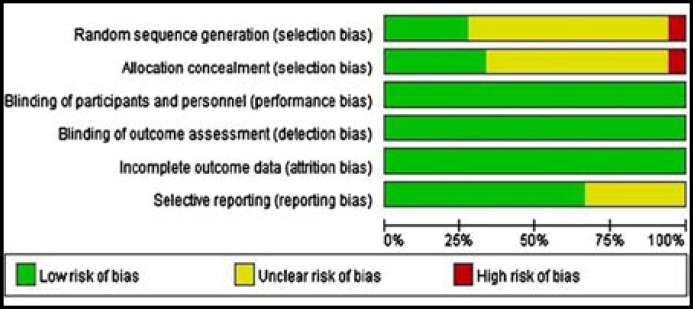
Risk of bias graph

**Fig.3:**
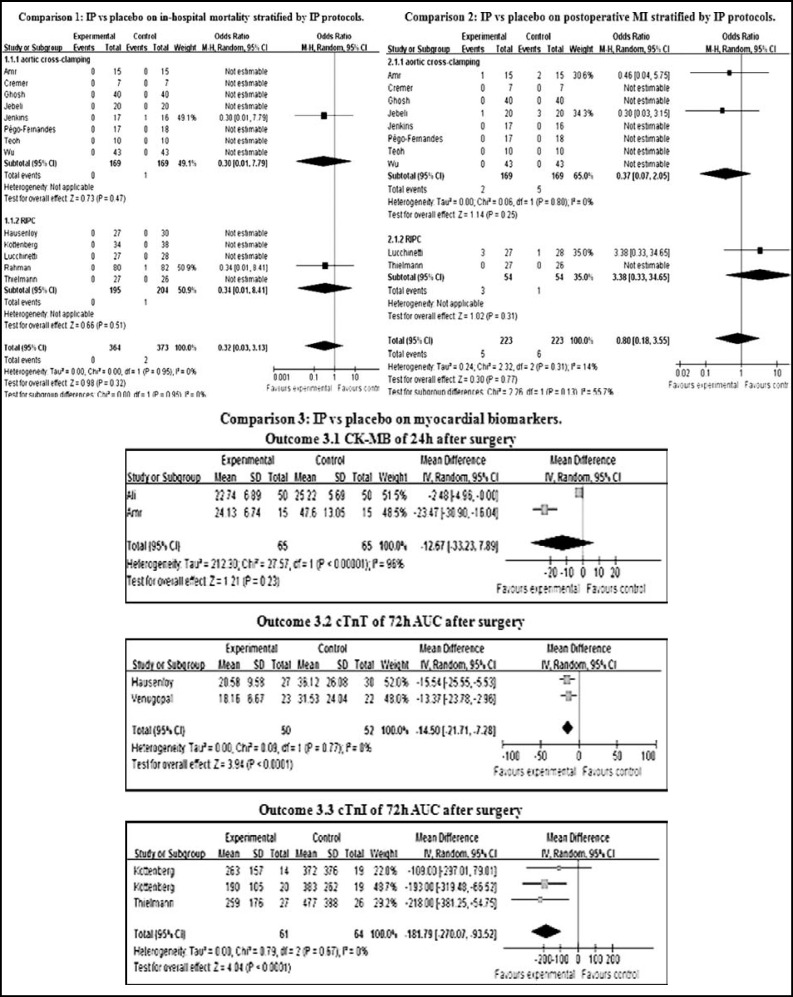
Forest plot (CI: Confidence interval; IP: ischemic preconditioning; RIPC: remote ischemic preconditioning; MI: myocardial infarction; ICCF: intermittent cross-clamp fibrillation; CK-MB: creatine kinase-MB; cTnT: cardiac troponin T; AUC: area under the curve; cTnI: cardiac troponin I).

**Table-1:** Characteristics of included studies (preconditioning/control groups).

*Study*	*Year*	*Patients number* *(female)*	*Mean age*	*vessel disease*	*Anesthesia* *(maintained by)*	*IP protocols*	*Timing of IP*	*Other myocardial protection*
Kottenberg ①	2012	14(5) /19(3)	65±15/64±12	3	propofol	3 cycles of 5-min left upper arm ischemia induced by a blood pressure cuff inflated to 200mmHg with an intervening 5-min of cuff deflation	after induction of anesthesia	hypothermia of 30–33°C, CBC
Kottenberg ②		20(1) /19(3)	64±9/ 65±9	3	isoflurane			
Lucchinetti	2012	27(1) /28(4)	59±7/62±10	2-4	isoflurane, opioids, rocuronium	4 5-min cycles of 300mmHg cuff inflation/deflation of the leg	before aortic cross-clamping	hypothermia (temperature was unclear), antegrade CBC
Karuppasamy	2011	27(5) /27(4)	66.9±11.2/67.3±10.3	2-5	isoflurane until CPB and after that with propofol	3 cycles of 5-min left upper arm ischemia induced by a blood pressure cuff inflated to 200mmHg with an intervening 5-min of cuff deflation	after anesthesia induction and before surgery	systemic hypothermia of 32℃, intermittent antegrade CBC(12/18), ICCF(15/9)
Jebeli	2010	20(8) /20(5）	48±7.8/45±5.8	no details	no details	2 cycles of 2-min ascending aortic clamping followed by 1-min reperfusion	after the initiation of CPB	antegrade and retrograde CBC, no hypothermia was induced
Rahman	2010	80(9) /82(10)	63/65	Multi-vessel	propofol, alfentanil; on CPB by enflurane or sevoflurane, propofol	3 cycles of 5-min upper-limb ischemia with 9-cm cuff inflation to 200mmHg separated by 5-min cuff deflation	after the initiation of CPB	intermitten antegrade CBC
Ali	2010	50(3) /50(8)	56.02±8.240/51.60±9.579	2-3	no details	3 cycles of 5-min forearm ischemia induced by cuff inflation to 200mmHg with an intervening 5-min of cuff deflation	after the anesthesia and before CPB	systemic hypothermia of 34℃, both antegrade and retrograde warm blood cardioplegia
Amr	2010	15(4) /15(2)	57±6/55±6	2-3	no details	3 cycles of 1-min aortic clamping followed by 4-min reperfusion	after the initiation of CPB, before CBC	hypothermia of 28-30℃, antegrade and retrograde CBC and a final warm blood cardioplegia (37℃) before aortic declamping
Thielmann	2010	27(4) /26(4)	63.4±11.3/64.1±12.3	3	either with isoflurane or propofol	3 cycles of 5-min left upper arm ischemia by inflation of a blood pressure cuff to 200 mmHg and 5-min reperfusion	after induction of anesthesia	mild systemic hypothermia (＞32℃), antegrade cold crystalloid cardioplegic
Venugopal	2009	23(4) /22(3)	62±9.7/64±9.0	1-4	either with halogenated anesthetics or propofol	3 cycles of 5-min right upper limb ischemia with cuff inflation to 200mmHg separated by 5-min cuff deflation	after anesthesia induction	intermittent antegrade and/or retrograde CBC
Hausenloy	2007	27(6) /30(6)	67±11.8/67±9.4	1-4	propofol	3 cycles of 5-min right upper arm ischemia induced by an automated cuff-inflator and inflated to 200mmHg with an intervening 5-min of cuff deflation	after anesthesia induction and before surgery	ICCF 17/18, cardiolegia 10/12
Buyukates	2005	10(3) /10(2)	61.2±5.2/63.2±4.0	2-3	nitrous oxide/ oxygen (50%/50%), isoflurane	2 cycles of 3-min aortic cross-clamping followed by 2-min reperfusion	after the initiation of CPB	antegrade CBC
Ghosh ①	2003	20/20	63.7±4.2/66.9±5.4	3	enflurane	1 cycles of 5-min aortic cross-clamping followed by 5-min reperfusion	immediately before the aortic cross-clamping	moderate systemic hypothermia (32°C), CBC, ICCF
Ghosh ②		20/20	63.8±5.9/61.4±9.2	3			before the first dose of cardioplegic solution	moderate systemic hypothermia (32°C), CBC
Wu	2002	43（10) /43(11)	63.7±1.4/66.9±1.4	3	no details	2 cycles of 2-min aortic clamping followed by 3-min reperfusion	after the initiation of CPB	antegrade and retrograde CBC, mild hypothermia (32℃)
Teoh	2002	10(2) /10(0)	64/65	3	propofo, midazolam; fentanyl was given as required	2 cycles of 3-min aortic cross-clamping followed by 2-min reperfusion	after establishing CPB, before the first graft was performed	ICCF
Pêgo-Fernandes	2000	17/18	no detail	≥ 2	no details	2 cycles of 3-min aortic clamping followed by 2-min reperfusion	before the standard operation	retrograde CBC, hypothermia(32℃) with intermittent aorta cross-clamping
Cremer	1997	7(1)/ 7(0)	62.1±4.6/58.1±4.6	3	no details	2 cycles of 5-min aortic clamping followed by 10-min reperfusion	after the initiation of CPB	intermitten antegrade CBC, moderate hypothermia (30℃)
Jenkins	1997	17(2) /16(1)	57/62	3	no details	2 cycles of 3-min aortic clamping followed by 2-min reperfusion	after the initiation of CPB	normothermia for first graft, and then moderte hypothermia of 32℃ thereafter, ICCF
Perrault	1996	10(3) /10(3)	68±3/63±4	no details	fentanyl, flunitrazepam, and isoflurane whenever required to adjust blood pressures	1 cycles of 3-min aortic clamping followed by 2-min reperfusion	after the initiation of CPB	continuous retrograde CBC, systemic hypothermia (31-32℃)

IP: ischemic preconditioning; CABG: coronary artery bypass graft surgery; CBC: cold blood cardioplegia; ICCF: intermittent cross-clamp fibrillation; CPB: cardiopulmonary bypass; LAD: left anterior descending; MI: myocardial infarction; LVEF: left ventricular ejection fraction; ECG: electrocardiograph

**Table-II:** Quality of evidence

*Outcomes*	Illustrative comparative risks* (95% CI)		*Relative effect(95% CI)*	*NO. of Participants(studies)*	*Quality of the evidence(GRADE)*
	*Assumed risk*	*Corresponding risk*			
	*Control*	*Ischemic preconditioning*			
in-hospital mortality	5 per 1000	2 per 1000 (0 to 17)	OR 0.32 (0.03 to 3.13)	737(13 studies)	⊕⊕⊕⊝moderate
postoperative MI	27 per 1000	22 per 1000 (5 to 89)	OR 0.80 (0.18 to 3.55)	446(10 studies)	⊕⊕⊝⊝low
CK-MB of 24h after surgery		The mean CK-MB of 24h after surgery in the intervention groups was 12.67 lower (33.23 lower to 7.89 higher)		130(2 studies)	⊕⊕⊝⊝low
cTnT of 72h AUC after surgery		The mean cTnT of 72h AUC after surgery in the intervention groups was 14.50 lower (21.71 to 7.28 lower)		102(2 studies)	⊕⊕⊝⊝low
cTnI of 72h AUC after surgery		The mean cTnI of 72h AUC after surgery in the intervention groups was 181.79 lower (270.07 to 93.52 lower)		125(3 studies)	⊕⊕⊝⊝low

CI: Confidence interval; OR: Odds ratio.

GRADE Working Group grades of evidence:

High quality: Further research is very unlikely to change our confidence in the estimate of effect. Moderate quality: Further research is likely to have an important impact on our confidence in the estimate of effect and may change the estimate.Low quality: Further research is very likely to have an important impact on our confidence in the estimate of effect and is likely to change the estimate.Very low quality: We are very uncertain about the estimate.

## METHODS

***Criteria for studies: ***Types of studies: All randomised controlled trials (RCTs) that compared IP with placebo in the presence or absence of other myocardial protection measures were eligible.

***Types of participants:*** Patients with on-pump coronary artery bypass graft surgery only, and aged 18 years or older.

***Types of interventions:*** Well described protocols of IP as the intervention.

***Types of outcome measures:*** Primary outcome was the in-hospital mortality. Secondary outcomes were postoperative myocardial infarction (MI), creatine kinase-MB (CK-MB) or cardiac troponin T/I (cTnT/I) level, and adverse effect.

***Search strategy: ***We searched the Cochrane Central Register of Controlled Trials (CENTRAL) on The Cochrane Library (Issue 3, 2013), the Medline/PubMed and CNKI in March 2013. All the articles published in English or Chinese were searched. The search terms were “ischaemic preconditioning”, “ischemic preconditioning”, “randomized controlled trial”, “random”, “controlled”.

***Selection of studies: ***Two reviewers independently reviewed the abstracts and selected trials which met the eligibility of criteria. Disagreement was discussed with a third reviewer. Then full texts of the articles were obtained for further evaluation.

***Data extraction and Assessment of risk of bias: ***Two reviewers extracted the data from trials and entered them into RevMan 5.1.6 for statistical analysis. Another reviewer checked the data. The quality assessment of included studies was done with GRADEprofiler 3.6.

***Data synthesis: ***Odds ration (OR) and ninety-five percent confidence interval (95% CI) were used for dichotomous variables. Mean differences (MD) were calculated for continuous variables. Probability values of P<0.05 were considered significant. Heterogeneity was evaluated using the chi-squared test with significance set at P≤0.10. When the included studies had enough similar, we took the Meta-analysis, and random effects model was used to pool data. Sensitivity analysis was used to test the reliability of the evidence. Publication bias and other biases were assessed by visual inspection of funnel plot only if there were more than 10 studies. 

## RESULTS

***Results of the search: ***We found 19050 studies. After removal of animal experiments, reviews, duplicates, and the studies which were not discussed the CAGB surgery, 46 RCTs were left. Through reading the titles and abstracts, we excluded 28 studies. Eighteen trials^4^^-^^21^ were ultimately included in our study (Fig.1).

***Description of studies: ***Eighteen RCTs with a total of 976 participants were included. They received on-pump CABG surgeries with other different myocardial protections, such as systemic hypothermia, antegrade and retrograde cold blood cardioplegia (CBC), intermittent cross-clamp fibrillation (ICCF). All researchers conducted IP after anesthesia and before standard operation. The IP protocols were aortic cross-clamping and limbs ischemia/reperfusion which induced by a cuff inflation and deflation. The characteristics of the studies were illustrated in Table-I.

***Risk of bias in included studies: ***The risk of bias is divided into three ranks according to the “Cochrane Handbook for Systematic Reviews of Interventions^22^”. Those are “high risk of bias”, “unclear risk of bias”, “low risk of bias”. Five studies^8^^,^^9^^,^^13^^,^^14^^,^^17^ provided the methods to generate randomization sequence (computer-generated randomization schedules). There was one study^6^ which used inappropriate method to include patients. The rest of the included studies didn’t give the methods for generation of allocation sequence in details. They just stated “randomization” instead. As the allocation concealment, six studies mentioned sealed envelops.^4^^,^^5^^,^^12^^-^^14^^,^^17^ IP was performed after the anesthesia which made the blinding of patients easily, so the performance bias was low. The outcomes of our study are objective indexes, so detection bias was low. All the outcomes were got before patients discharged, so no data was lost (Fig.2).

***Effects of interventions: ***Considering the clinical heterogeneity between studies, we took the subgroup analysis, examined the effect of IP versus control stratified by IP protocols (aortic cross-clamping, RIPC). When there was no heterogeneity for the outcomes, a random-effect model was used to pool the data**.**

***Effect of IP on in-hospital mortality: ***13 studies reported the in-hospital mortality,^5^^,^^7^^-^^11^^,^^13^^-^^15^^,^^17^^-^^19^^,^^21^ 364 participants were performed IP and 373 participants were in control groups. Two participants died, both of them were in control groups. One died of multiple organ failure which developed from a peptic ulcer and then septicemia.^11^ One caused by pneumonia.^17^ There was no heterogeneity among trials stratifying by IP protocols. (X^2^=0.00, P=0.95). Random-effect meta-analysis showed no difference on in-hospital mortality between the two groups (OR=0.32, 95%CI 0.03 to 3.13).

***Effect of IP on postoperative MI morbidity: ***Ten studies provided information on postoperative MI,^5^^,^^7^^,^^8^^,^^10^^,^^11^^,^^14^^,^^15^^,^^18^^,^^19^^,^^21^ 223 participants were in experimental groups (five got MI) and 223 participants were in control groups (six got it). There was no heterogeneity among trials stratifying by IP protocols. (X^2^=2.32, P=0.31, I^2^=14%). Random-effect meta-analysis showed no difference on postoperative MI morbidity between the two groups (OR=0.80, 95%CI 0.18 to 3.55).

***Effect of IP on myocardial biomarkers: ***Two studies reported the CK-MB level 24 hours after surgery.^4^^,^^5^ But heterogeneity was obvious between IP groups and control groups (X^2^=27.57, P＜0.000001, I^2^=96%). No difference was observed via random effects model (MD=-12.67, 95%CI -33.23 to 7.89). There were other four studies which provided the CK-MB information at different time points. In all these six studies, three^4^^-^^6^ supported a cardioprotective effect of IP for CABG while the other three^7^^,^^15^^,^^16^ were not. Cremer^7^ even reported that IP seemed to impair the contractile function inversely. 

Two studies provided information on the 72 hours area under the curve (AUC) of cTnT after surgeries.^9^^,^^20^ There was no heterogeneity (X^2^=0.09, P=0.77), and difference was observed (MD=-14.50, 95%CI -21.71 to -7.28). Other two studies which reported cTnT level gave the positive conclusions,^11^^,^^18^ while other four studies made the negative conclusions.^7^^,^^8^^,^^14^^,^^17^

Two studies gave the information about the 72 hours AUC of cTnI after surgeries.^13^^,^^19^ No heterogeneity was observed (X^2^=0.79, P=0.67), and there was a significant difference between groups (MD=-181.79, 95%CI -270.07 to -93.52). There was another study reported cTnI made the positive conclusion.^5^ Yet other two studies gave the negative conclusions.^12^^,^^15^ All the analysis showed in forest plot (Fig.3).

## DISCUSSION

***Summary of main results: ***Based on the pooled data, IP may not have a positive effect on reducing in-hospital mortality, postoperative MI and plasma CK-MB level, but may decrease cTnT/I level. All of the 18 RCTs, the positive results accounted for half, and the negative results too. So these meta-analysis results should be interpreted with caution due to limited effective data.

***Quality of the evidence: ***Most of the included studies didn’t provide the specific description of the randomization procedure, and allocation concealment was unclear. These could lead to selection bias. Although blinding was unclear in many included studies, the intervention was carried out after the anesthesia and the outcomes were objective indexes, so the risk of performance and detection bias would be low which may not contribute to false positive or negative results. All outcomes could get after operations before patients discharged, so there would not have incomplete data. IP is easy to perform and requires little special equipment, no obvious adverse effect was reported, and so interest conflicts are less. It was impossible to perform a funnel plot for publication bias because of the small numbers of studies with effective data. The total quality of evidence see Table-II.

***Implications for practice: ***During the CABG surgery procedures, there are many potential confounding variables. Hu^23^ studied the function of emulsified isoflurane for myocardial IRI in rats and the results supported the protective effects. Kottenberg and coworkers^13^ reported RIPC with isoflurane anesthesia attenuated myocardial injury in patients underwent CABG but not propofol. These means that different anesthesia methods, even some other kinds of medicines could cause pharmacological preconditioning.

The timing of IP, some researchers performed it after cardiopulmonary bypass (CPB), while others after anesthesia, is different. Di Salvo C and associates^24^ reported that the ATP levels in patients took preconditioning and kept normothermia during ventricular fibrillation is lost when patients took similar preconditioning were cooled to 32℃. According with their theory that interventions which could reduce myocardial oxygen consumption after ischemia may prevent the protective function of preconditioning from hypothermia. 

As for the CPB, it could induce a systemic inflammatory reaction, generate free radical specie.^25^ Therefore it may cause the cardioprotection. Some researchers also believe that CPB has certain form of preconditioning through activation of adenosine and ɑ-adrenergic receptors.^26^

Most of the animal experiments made positive conclusions, while it was not in human beings. Perrault and coworkers^27^ considered that, any additional myocardial protection methods were used in animal heart ischemic model, while in humans’ heart surgeries were used routinely, such as cold/ warm cardioplegia, systemic hypothermia, intermittent cross-clamp fibrillation (ICCF). There is a great difference between animal ischemia models and humans’ cardiac surgery. 

Because of these clinical heterogenity, although there are some negative results, that could not make IP be denied completely.

***Implications for research: ***IP is easy to perform, requires little special equipment. It is likely to be high cost-effectiveness. Considering the potential confounding variables exist, if authors want to testify IP’s effort, multi-centre, randomized, double-blind, placebo-controlled studies are needed, and the operation procedures and included criteria should be more specific. The following features should be addressed in future studies.

- Correct randomization sequence generation methods and allocation concealment procedures should be used and reported in detail.
- Application of blinding. Let one person conduct the IP procedures who is not involved in the studies.
- Operation procedures and included criteria should be more specific, such as types of operations, anesthesia drugs, IP protocols, timing of IP, additional myocardial protection methods.
- Baseline characteristics and Routine treatments should be described in detail.
- Adverse events are always needed to be reported.
